# Enhanced Acquisition and Retention of Conditioned Eyeblink Responses in Veterans Expressing PTSD Symptoms: Modulation by Lifetime History of Mild Traumatic Brain Injury

**DOI:** 10.3389/fnbeh.2020.595007

**Published:** 2020-12-08

**Authors:** Justin D. Handy, W. Geoffrey Wright, Amanda Haskell, Labeeby Servatius, Richard J. Servatius

**Affiliations:** ^1^Department of Veterans Affairs, Syracuse Veterans Affairs Medical Center, Syracuse, NY, United States; ^2^Central New York Research Corporation, Syracuse, NY, United States; ^3^Neuromotor Sciences Program, College of Public Health, Temple University, Philadelphia, PA, United States; ^4^Department of Psychiatry, State University of New York Upstate Medical University, Syracuse, NY, United States

**Keywords:** PTSD, mild traumatic brain injury, depression, military personnel, temperament, personality

## Abstract

Enhanced acquisition of eyeblink conditioning is observed in active duty military and veterans expressing PTSD symptoms (PTSD^+^) and those expressing temperamental vulnerabilities to develop PTSD after traumatic experiences, such as behaviorally inhibited temperament. There is a growing literature showing persistent cerebellar abnormalities in those experiencing mild traumatic brain injury (mTBI^+^) as well as linkages between mTBI and PTSD. With the dependency of eyeblink conditioning on cerebellar processes, the impact of mTBI on eyeblink conditioning in veterans expressing PTSD is unknown. The present study assessed eyeblink conditioning in veterans during two sessions separated by 1 week. With a focus on the accelerated learning of veterans expressing PTSD, training utilized a protocol which degrades learning through interspersing conditioned stimulus (CS) exposures amongst delay-type trials of CS and unconditional stimulus (US) co-terminating trials. Faster acquisition of the eyeblink conditioned responses (CR) was observed in PTSD during Week 1. The Week 2 assessment revealed an interaction of mTBI and PTSD, such that asymptotic performance of PTSD^+^ was greater than PTSD^−^ among mTBI^−^ veterans, whereas these groups did not differ in mTBI^+^ veterans. To further examine the relationship between enhanced sensitivity to acquire eyeblink conditioning and PTSD, cluster analysis was performed based on performance across training sessions. Those with enhanced sensitivity to acquire eyeblink conditioned responses expressed more PTSD symptoms, which were specific to Cluster C symptoms of avoidance, in addition to greater behavioral inhibition. These results support the continued investigation of the conditioned eyeblink response as a behavioral indicator of stress-related psychopathology.

## Introduction

Diathesis models elaborate interactions between inherent vulnerabilities and experiential risk factors in the development of stress-related mental health problems, such as post-traumatic stress disorder (PTSD). PTSD is currently defined by symptom expression in four clusters: intrusive thoughts, avoidance, negative mood, and heightened arousal and reactivity (Lee et al., 2019). For PTSD, a learning diathesis model is proposed as a proxy for understanding the development and persistence of acquired changes in behavior and coping in the aftermath of trauma experience represented by intrusion and avoidance symptoms, in particular, and indirectly by arousal and enhanced reactivity symptoms (Allen et al., 2016, 2019; Servatius, 2016).

A basic assumption of a learning diathesis model of PTSD is deviant associative learning reflects stable learning biases that predate trauma exposure, rather than trauma-derived changes in associative learning power or processing. Eyeblink conditioning, a simple form of classical conditioning, provides a convenient methodology for assessing inherent learning biases in PTSD inasmuch as the critical stimuli used in the assessment of eyeblink conditioning are relatively independent of trauma occurrence. Eyeblink conditioning encompasses acquisition of an eyeblink conditioned response (CR) through successive pairing of a conditioned stimulus (CS; e.g., sound) with an unconditional stimulus (US; a mild puff of air to the eye) inducing an unconditional response (UR; a reflexive eyeblink). As an associative learning paradigm, eyeblink conditioning has high translational value for neurologic mental health influences (Steinmetz et al., 2001; Greer et al., 2005; Edwards et al., 2008; Myers et al., 2012b; Greer and Thompson, 2017; Handy et al., 2018) with protocols validated for humans and non-human mammals (e.g., Servatius, 2000) and strong evidence of neural substrates conserved across mammalian species (Miller et al., 2003; Cheng et al., 2008; Freeman and Steinmetz, 2011).

Evidence supporting the predating assumption, although difficult to derive in humans, is more apparent in animal models. Exposure to intense inescapable stressors designed to mimic trauma facilitated acquisition of eyeblink conditioning in male rats (Servatius and Shors, 1994; Beck et al., 2002). However, the facilitatory biases are effervescent, dissipating within days of trauma exposure (Shors and Servatius, 1997; Beck et al., 2002; Servatius and Beck, 2003; Servatius et al., 2005). One source of stable associative learning biases predating trauma is linked to personality in the form of behaviorally inhibited (BI) temperament. BI temperament, defined by extreme withdrawal in the face of novel social and non-social stimuli, is identified with PTSD and other anxiety disorders (Kagan et al., 1987; Biederman et al., 1990; Hirshfeld et al., 1992; Gladstone and Parker, 2005; Morgan, 2006; Degnan and Fox, 2007; Gudino, 2013). Associative learning biases are apparent in BI, with faster eyeblink conditioning expressed in adolescents (Holloway et al., 2012, 2014; Caulfield et al., 2013, 2015; Allen et al., 2014), college students (Allen et al., 2014), active duty military (Handy et al., 2018), and veterans (Myers et al., 2012b). In active duty military (Servatius et al., 2017) and veteran samples (Myers et al., 2012a), BI was also strongly associated with self-reported PTSD symptoms.

Facilitated acquisition could be the product of enhanced processing of the CS pathway (i.e., pontine nuclei to deep cerebellar nuclei and cerebellar cortex through mossy fibers), US pathway (i.e., inferior olive to deep cerebellar nuclei and cortex through climbing fibers) or the conjunction. One means to infer the nature of the learning bias is through manipulation of the ratio of CS to US during conditioning. Allen et al. (2014) compared acquisition of the eyeblink CR in BI and non-BI college students in two degraded contingencies: a training schedule that the CS was followed by the US on only 50% of trials (i.e., a partial reinforcement training schedule) and a schedule in which the US was preceded by the CS on 50% of trials (i.e., a partially-predictable reinforcement schedule). The former examined sensitivity to the presence of the cue, whereas the latter tested sensitivity to the aversiveness to the US. In both protocols, models of associative strength predict degraded learning under moderate stimulus intensity levels compared to continuous reinforcement schedules. Facilitation was apparent under both degraded contingencies; that is, the BI group performed better with each degraded contingency. Interestingly, a comparison of the learning of the non-BI group showed degraded learning was only apparent in the degraded CS condition; learning with 50% of the USs during training resulted in equivalent learning in BI and non-BI compared to the 100% paired training. An important implication of the study was sensitivity to the learning bias was enhanced with the degraded contingencies; facilitated acquisition was apparent with far fewer participants, roughly half to a third of those participating in previous studies. This is an important consideration for assessment of smaller populations or occupational groups in applied settings which volunteerism is more modest than that of undergraduate institutions, such as active duty military and veteran populations.

A complication in understanding the role of associative learning biases in PTSD, particularly for cerebellar-based behavioral probes, is the possible moderating effect of mild traumatic brain injury (mTBI). Active duty service members are at a higher risk to experience a mTBI event during service (Bazarian et al., 2005; Hoge et al., 2008), which compounds risk for re-injury as a civilian (Carlson et al., 2012). Although mTBI was long thought to represent a transient, otherwise benign alteration in consciousness for the vast majority of those experiencing mTBI, more recent evidence finds enduring brain alterations in larger proportions of those experiencing mTBI. The effects of mTBI on the cerebellum are less appreciated, although a growing literature highlights cerebellar structural and functional changes following mechanical and blast injury events (Sato et al., 2001; Gale et al., 2005; Meabon et al., 2016), including evidence linking cerebellar dysfunction to cerebellar pathology in active duty military and veterans (Mac Donald et al., 2011, 2013; Peskind et al., 2011; Petrie et al., 2014; Meabon et al., 2016). Given the dependency of eyeblink conditioning on cerebellar processing, mTBI is a potential complication in understanding the relationship of associative learning as indexed by eyeblink conditioning to PTSD. Further, lifetime experience of mTBI appears to negatively affect auditory reactivity in active duty military personnel (Wright et al., 2018). Although mTBI was not a significant contributor in Coast Guard eyeblink conditioning study cited previously (Handy et al., 2018), there were suggestions of degraded learning among those reporting lifetime history of mTBI (unpublished observations). However, the number of Coast Guard personnel with lifetime mTBI and expressing PTSD who also participated in the eyeblink conditioning assessment was too few to clearly evaluate among subgroups.

The current study assessed eyeblink conditioning in veterans currently experiencing PTSD symptoms using a degraded contingency of paired trials (i.e., CS+ trials) interspersed with CS-alone trials (i.e., CS– trials). Recent evidence in university samples and active duty military suggests partial reinforcement training schedule may maximize observable differences in learning between symptom groups. It was expected that those expressing PTSD symptoms would exhibit faster acquisition of the eyeblink CR under the degraded contingency. Further, the facilitative learning effects associated with PTSD were expected to be less pronounced in those with lifetime experience of mTBI. The moderating influence of mTBI on learning in PTSD populations has not been formally examined to date in eyeblink conditioning. As prevalence rates for mechanical force and blast-related mTBI are exceptionally high among veterans, accounting for variability in conditioned responding owed to brain injury is critical.

Training in the current study occurred over two sessions separated by a week. The second training session served several purposes: First, repeated training in eyeblink classical conditioning in humans is rarely assessed. The second session thus provided the opportunity to assess retention of acquired responses and provided additional insight into acquisition. Second, the second session provided the opportunity to assess the stability of acquired responses. In previous studies, conditioned responding in BI and PTSD demonstrated a characteristic delay to extinguish (Allen et al., 2014; Handy et al., 2018); that is, when the CS was presented in the absence of the US over trials, the eyeblink CR persisted in PTSD and anxiety-prone individuals to a greater degree than in their respective comparison groups. To date, no study has examined the stability of the eyeblink CR in humans over a protracted period of time, independent of experimental manipulations to extinguish the response. The question of retention is critical insofar as the classically conditioned eyeblink response has the potential to serve as a behavioral biomarker for anxiety and stress disorders that could be used to inform mental health professionals in early identification, treatments, and therapeutics.

There is now substantial evidence that those self-reporting BI and/or PTSD+ are likely as a group to acquire eyeblink conditioned responses to a higher degree than NI or PTSD-. Is the converse also true? That is, are those who acquire eyeblink conditioning to a greater degree more likely to self-report symptoms? The suboptimal learning protocol provides the means to better distinguish learning. With these considerations in mind, the relationship between enhanced sensitivity to acquire eyeblink conditioning and PTSD was further examined in an exploratory cluster analysis focused on learning. Unique learning profiles were constructed using block-by-block eyeblink conditioning performance data as inputs. The resulting groups were compared on self-report measures of current PTSD symptomology and related psychological factors. Critically, the unsupervised clustering procedure aggregates data points together independent of known outcomes. In other words, learning profiles were developed solely on the basis of expression of the eyeblink CR. It was expected that those exhibiting a higher degree of asymptotic performance of the eyeblink CR under degraded contingency would express PTSD symptoms and related psychological factors to a greater degree than those exhibiting relatively poorer asymptotic performance.

## Materials and Methods

### Participants and Recruitment

Study volunteers included 60 military service veterans served at the Syracuse VA Medical Center (SVAMC, Syracuse, NY). Male and female veterans between the ages of 18 and 65 were eligible to participate. Exclusionary criteria included history of hearing impairment and current experience of symptoms related to schizophrenia and/or bipolar mood disorder. Participants were recruited using printed advertisements posted within the SVAMC and associated community clinics. Veterans interested in participating were instructed to contact the study staff via telephone, at which time they were given a brief study description and administered pre-screening measures to determine study eligibility. All eligible participants completed an informed consent agreement upon arrival for their scheduled study appointments and were given the opportunity to ask questions before initiating study procedures. Participants were compensated $50 for each completed study session for a total of $100. Payment was in the form of a check mailed to an address designated by the participant. This study was reviewed and approved by the Syracuse/Bath/Canandaigua VA Medical Centers Institutional Review Board (IRB). All research was conducted in compliance of the Declaration of Helsinki and all subsequent amendments.

### Self-Report Measures

All participants completed a battery of self-report measures during their first session. This assessment battery included measures of current post-traumatic stress symptoms, current depressive symptoms, concussion history, and an assessment of BI temperament. Detailed descriptions of these measures, including scoring criteria, are included below:

#### Post-traumatic Stress Symptoms

The Post-traumatic Stress Disorder Checklist for DSM-5 (PCL-5; Weathers et al., 2013) is a 20-item survey corresponding to the current DSM-5 criteria for the diagnosis of PTSD. Respondents report the degree to which they have been bothered by post-traumatic symptoms over the past month using a 5-point Likert scale, with responses ranging from 0 = “Not at all” to 4 = “Extremely.” Determination of a provisional PTSD diagnosis was based on the recommended cut-off of 33 for the total symptom severity score corresponding to the sum of scores for each of the 20 items (range 0–80).

#### Depressive Symptoms

The Patient Health Questionnaire-8 (PHQ-8; Kroenke et al., 2009) was used to assess how often depressive symptoms were bothersome over the last 2-weeks period. Occurrence was rated on a 4-point Likert scale that included responses “Not at All,” “Several Days,” “More Than Half the Days,” and “Nearly Every Day.” Deriving positive screens followed two approaches: symptom scoring, which required endorsement of either depressed mood or anhedonia “More Than Half the Days” and at least 5 of the 8 symptoms to be present “More Than Half the Days,” and aggregate scoring, which classified major depressive disorder symptomology as None (0–4), Mild (5–9), Moderate (10–14), Moderately Severe (15–20), and Severe (>20), with a score of 10 or more as indicative of clinically significant MDD.

#### Concussion History

The DVBIC TBI Screening Tool (Terrio et al., 2011; Schwab et al., 2015) was used to assess present/lifetime mTBI status. Verbally and individually administered with each participant, the screening tool determined whether the participant experienced a head injury, the nature of the head injury, whether the participant lost consciousness and for how long, and the degree to which current symptoms are attributable to head injury.

#### BI Temperament

The Adult Measure of Behavioral Inhibition (AMBI; Gladstone and Parker, 2005) consists of 16 items probing aspects of BI temperament. Items assess the degree to which behaviors are exhibited in social and non-social situations on a 3-point Likert scale (ranging from ‘No/hardly ever’ to ‘Yes/most of the time’). Total scores range from 0 to 32. Those with scores above 15.5 were classified as BI.

### Conditioning Apparatus and Procedure

The procedures for eyeblink conditioning followed those previously defined (Allen et al., 2014; Handy et al., 2018). Auditory stimuli were produced by software signal generators in MATLAB and a digital-to-analog converter (USB-6211, National Instruments) and passed through a David Clark aviation headset (Model H10-50, Worchester, Massachusetts, USA). A Realistic sound meter (Radio Shack, Fort Worth, Texas, USA) was used to verify sound levels. The headphones will provide the auditory stimuli for eyeblink conditioning (82 dB pure tone, 500-ms, 5-ms rise/fall). Participants watched a silent movie (e.g., Pixar animated shorts) to maintain attention throughout the conditioning session. Air puffs were produced by pressurizing ambient air to 5-psi (e.g., Fürgut Industries, Aitrach, Germany), and released through silastic tubing attached to the boom of the headphones by a computer-controlled solenoid valve (e.g., Clipper Instruments, Cincinnati, OH). The boom was placed ~1-cm from the eye and aimed at it.

Eyeblink responses were obtained through electromyography (EMG) signal recording via surface silver/silver chloride electrodes coated with a conductive gel. These electrodes were placed above and below the right eye, with a ground sensor placed on the forehead. A BMA-200 isolated physiological amplifier (CWE, Ardmore, Pennsylvania, USA) was used to electronically band-pass (1–30 Hz) and amplify the signal by a factor of 1,000. The resulting signal was sampled at 1,000 Hz and digitized through an analog-to-digital converter board (USB-6211, National Instruments).

A partial reinforcement schedule was utilized in which a 500-ms pure tone conditioned stimulus (CS; e.g., 1,200 Hz at 82 dB) co-terminated with a 50-ms air puff unconditional stimulus (US) on 50% of trials (CS+); for the remaining 50% of trials, participants were exposed to the CS alone (CS-). Trial type was pseudorandom such that no more than 3 CS+ or CS– trials occurred in succession. The first conditioning session began with three US-alone exposures to establish UR quality and magnitude of response. The training session then consist of 60 acquisition trials. The inter-trial interval will be 15–30 s. The eyeblink conditioning protocol for Week 1 took ~45 min to complete, including time taken for instrumentation for EMG and administration. Following completion of the Week 1 protocol, participants were dismissed and instructed to return 1 week later for the second session.

Upon returning 1 week later, participants were re-instrumented for eyeblink conditioning and completed a modified protocol that included 30 conditioning trials under a 50% partial reinforcement schedule. No US-alone exposures were included in the Week 2 protocol.

### Signal Processing

EMG data were evaluated on a trial-by-trial basis for all participants using a 3,000-ms sampling period for each trial. A defined time window for the CR was set at 80-ms after CS onset, but prior to the US onset for paired trials. To determine the occurrence of an eyeblink, a threshold value of 0.2 (unitless) was used as criterion for the peak detection function. If a peak was detected but did not exceed the threshold or a 250-ms mean of the baseline plus two standard deviations, the peak was not counted as an eyeblink response. The slope coefficients (COS 1/COS 2), representing the forward and backward facing slope of the eyeblink waveform ±25-ms on either side of the peak, were used as additional criteria for a detected peak to be counted as a proper response. To avoid being counted as a false positive identification of an eyeblink response, the slope of the detected wave had to be sufficiently high to resemble a typical eyeblink.

### Analytic Approach

All statistical analyses were performed in IBM SPSS Statistics for Windows, version 26 (IBM Corp., Armonk, NY, USA) and R (R Core Team, 2020). For analysis of eyeblink conditioning data, separate mixed factor analysis of covariance (ANCOVA) models were constructed for Week 1 and Week 2 performance using the percentage of conditioned eyeblink responses in each trial block as repeated measures, PTSD and mTBI as independent variables, and age as a covariate. The inclusion of age as a covariate was based on a substantial literature demonstrating deficits in acquisition of conditioned eyeblink responses in delay conditioning protocols attributable to normal aging processes (e.g., Solomon et al., 1988; Woodruff-Pak and Thompson, 1988). All repeated measures data were corrected for violations of the sphericity assumption using a Greenhouse-Geisser correction. All *post-hoc* comparisons were performed using Bonferroni-corrected p-values to account for the increased risk of Type I error following multiple comparisons.

Finally, an exploratory cluster analysis was performed in which veterans were classified on the basis of acquisition of conditioned eyeblink responses and the resulting behavioral profiles were compared against relevant mental health outcomes. For data reduction in the current study, an agglomerative hierarchical clustering approach was adopted using squared Euclidean distance as the dissimilarity measure and a complete (or furthest neighbor) linkage measure. In this hierarchical clustering procedure, veterans initially occupied independent clusters in the first step of the analysis, with subsequent steps combining veterans into homogenous groups on the basis of the average frequency of CRs expressed in each training block. This procedure continued until all veterans were grouped into a single cluster. To determine the optimal number of clusters, the “NbClust” R package was utilized with default settings (Charrad et al., 2014). This algorithm used 30 unique indices to determine the optimal clustering solution through majority rule. Having established distinctive learning profiles, differences in mental health status and personality were assessed for each group using t-tests or univariate ANOV, as dictated by the number of groups produced by the cluster analysis.

## Results

### Sample Characteristics

Of the 60 veterans that volunteered for the study, data for six participants was excluded from analysis. Four participants failed to complete the eyeblink conditioning training session due to equipment failure and could not be rescheduled, whereas an additional two participants were excluded based on self-report of multiple sclerosis (MS). MS has been shown to adversely affect acquisition of the conditioned eyeblink response (Rampello et al., 2011). Descriptive statistics for the remaining 54 participants are presented in Table 1. Participants were grouped according to PTSD and mTBI designation.

**Table 1:** Descriptive statistics for demographic and self-report measures as a function of PTSD and mTBI classification.

	**PTSD^**−**^/mTBI^**−**^ (*n* = 8)**	**PTSD^**−**^/mTBI^**+**^ (*n* = 21)**	**PTSD^**+**^/mTBI^**−**^ (*n* = 12)**	**PTSD^**+**^/mTBI^**+**^ (*n* = 13)**
Age	46 (11.50)	51 (10.00)	50 (10.50)	45 (12.00)
Sex (M/F)	6/2	18/3	10/2	9/4
PCL-5	10.00 (6.63)	19.19 (8.52)	47.67 (11.24)	51.46 (9.70)
PHQ-8	7.50 (4.31)	5.57 (3.81)	14.92 (7.13)	12.46 (4.37)
AMBI	16.63 (6.68)	16.91 (5.70)	20.25 (6.37)	22.08 (6.86)

### Eyeblink Conditioning

**Figure 1:**
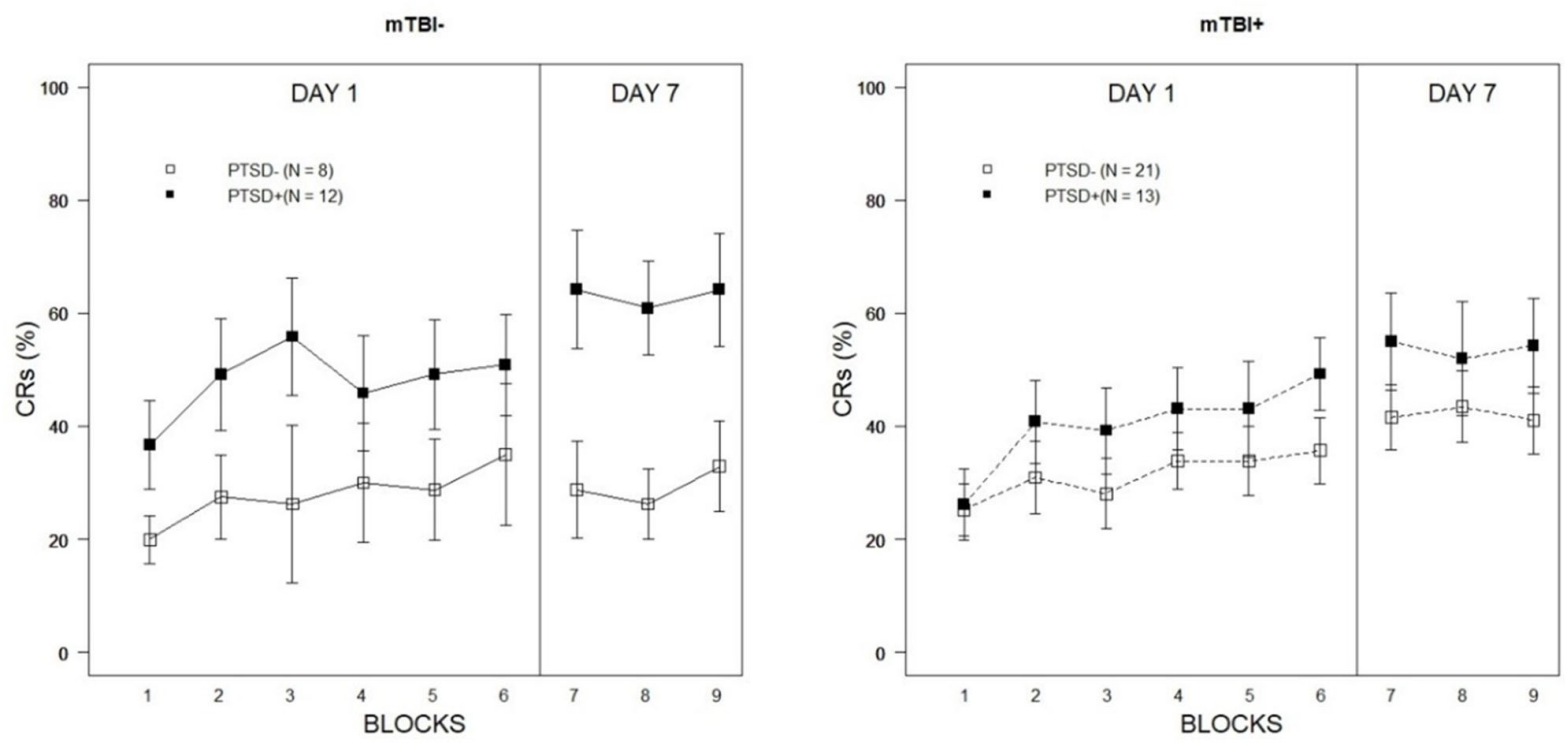
Percentage of eyeblink CRs expressed over Week 1 and Week 2 training blocks as a function of PTSD classification and lifetime history of mTBI. Error bars represent ±1 standard error of the mean.

The Week 1 training session was analyzed with a 2 × 2 × 6 (PTSD × mTBI × Training Block) mixed-ANCOVA with age entered as a continuous covariate. Mauchly's test indicated that the assumption of sphericity had been violated for the repeated measure [$X_{\left(14\right)}^{2}$ = 27.51, *p* = 0.017], therefore Greenhouse-Geisser estimates of sphericity (ε = 0.83) were used to correct the degrees of freedom for the repeated measures analysis. There was a significant main effect of Training Block, confirming the expected acquisition of eyeblink CRs over the course of the training session [*F*_(4.16,203.75)_ = 2.92, *p* = 0.013, partial η^2^ = 0.06]. There were no interaction effects apparent between Training Block and PTSD [*F*_(4.16,203.75)_ = 0.61, *p* = 0.662, partial η^2^ = 0.01), Training Block and mTBI [*F*_(4.16,203.75)_ = 0.35, *p* = 0.850, partial η^2^ = 0.01], nor a Training Block × PTSD × mTBI interaction [*F*_(4.16,203.75)_ = 0.32, *p* = 0.870, partial η^2^ = 0.01]. However, as shown in Figure 1, there was a main effect of PTSD [*F*_(1,49)_ = 4.66, *p* = 0.036, partial η^2^ = 0.09]. In general, PTSD+ veterans performed better than PTSD– veterans. The main effect of mTBI was not significant [*F*_(1,49)_ = 0.25, *p* = 0.619, partial η^2^ = 0.01], nor was the interaction between mTBI and PTSD [*F*_(1,49)_ = 2.87, *p* = 0.096, partial η^2^ = 0.06].

Week 2 training performance was evaluated with a 2 × 2 × 3 (PTSD × mTBI × Training Block) mixed-ANCOVA with age again entered as a continuous covariate. The main effect of Training Block was not significant [*F*_(2,98)_ = 0.42, *p* = 0.658, partial η^2^ = 0.01]. Similarly, there was no indication that Training Block interacted with PTSD [*F*_(2,98)_ = 0.13, *p* = 0.878, partial η^2^ = 0.00], mTBI [*F*_(2,98)_ = 0.43, *p* = 0.654, partial η^2^ = 0.01], nor was the three-way interaction between Training Block, PTSD, and mTBI significant [*F*_(2,98)_ = 0.27, *p* = 0.765, partial η^2^ = 0.01]. The main effect of PTSD was significant [*F*_(1,49)_ = 9.19, *p* = 0.004, partial η^2^ = 0.16] but was qualified by a significant PTSD × mTBI interaction [*F*_(1,49)_ = 5.55, *p* = 0.022, partial η^2^ = 0.10]. As illustrated in Figure 1, the facilitative effects of current post-traumatic symptoms were far more apparent among veterans without a history of mTBI. Among those veterans with a history of mTBI, there were no differences in the percentage of CRs expressed during Week 2 as a function of current PTSD symptoms [*t*_(32)_ = 0.46*, p* = 0.65]; in contrast, PTSD+ veterans without a history of mTBI expressed a significantly higher CR percentage than PTSD– veterans during Week 2 testing [*t*_(18)_ = 3.43, *p* = 0.003]. These results suggest mTBI may have a moderating effect on the stability of learning biases associated with PTSD.

### Behavioral Subtyping Analysis

**Figure 2:**
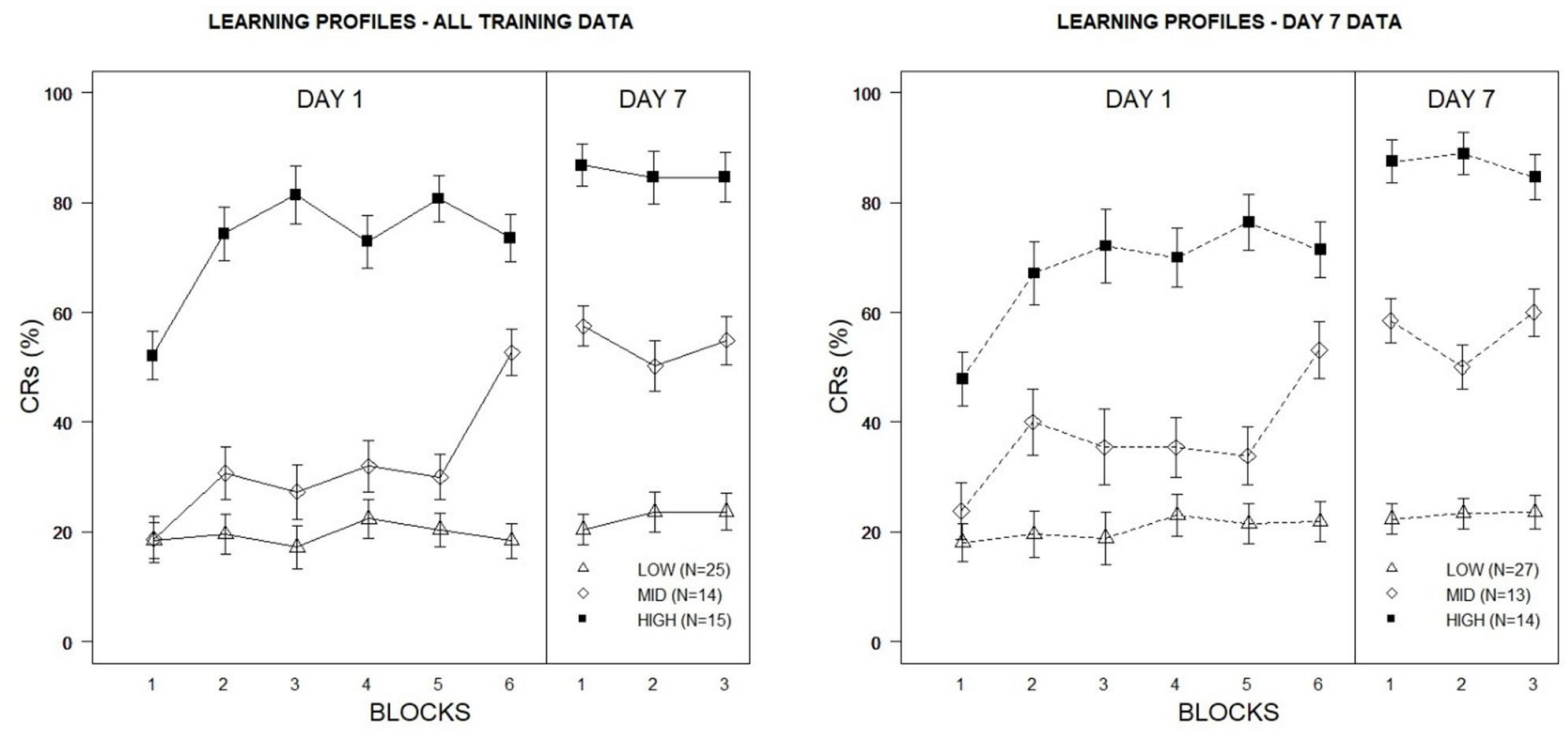
Percentage of eyeblink CRs for Veterans based on learning profiles derived from cluster analyses of eyeblink conditioning acquisition data. The left figure depicts high, mid, and low learning profiles constructed using Day 1 and Day 7 conditioning data. The right figure depicts high, mid, and low learning profiles constructed using Day 7 conditioning data only. Error bars represent ±1 standard error of the mean.

The hierarchical cluster analysis resulted in a three-group solution to optimally distinguish veterans on the basis of eyeblink conditioning performance, using the proportion of eyeblink CRs across Week 1 and Week 2 training blocks as inputs. 11/30 indices included in the NbClust R package agreed with the 3-group solution. Based on the distinctive acquisition profiles shown in Figure 2 (left panel), veteran learning was characterized as High (*n* = 14/54; 26%), Mid (*n* = 15/54; 28%), and Low (*n* = 25/54; 46%). The three learning groups boasted unique acquisition profiles in that High Learners expressed a high frequency of eyeblink CRs very early in training, whereas Mid Learners showed a more gradual acquisition rate over time. Low Learners demonstrated minimal acquisition of the eyeblink CR under the degraded learning conditions imposed by the 50% CS partial reinforcement training schedule.

Of interest was to what degree these learning groups would differ in terms of personality and psychopathological factors. Descriptive statistics for these measures as a function of learning groups are presented in Table 2. Group difference analyses on self-report measures revealed significant group differences in total PCL scores [*F*_(2,51)_ = 7.58, *p* = 0.001, partial η^2^ = 0.23], total PHQ-8 scores [*F*_(2,51)_ = 6.71, *p* = 0.003, partial η^2^ = 0.21], as well as total AMBI scores [*F*_(2,51)_ = 4.18, *p* = 0.021, partial η^2^ = 0.14] assessing BI. A follow-up analysis focusing on PTSD symptomology examined group differences on the basis of PCL Cluster B, C, D, and E scores. Significant group differences were evident for Cluster C [*F*_(2,51)_ = 8.27, *p* = 0.001, partial η^2^ = 0.25], Cluster D [*F*_(2,51)_ = 3.46, *p* = 0.039, partial η^2^ = 0.12], and Cluster D [*F*_(2,51)_ = 3.44, *p* = 0.04, partial η^2^ = 0.12]. Helmert contrasts revealed significant differences between the Low Learners and the more proficient learning groups for Cluster C (*p* < 0.0001), Cluster D (*p* = 0.012), and Cluster E (*p* = 011) symptom scores. There were no significant differences between Mid and High Learners for Cluster C (*p* = 0.590), Cluster D (*p* = 0.739) nor Cluster E (*p* = 0.950) symptom scores. Group differences in Cluster B symptoms did not reach statistical significance [*F*_(2,51)_ = 3.09, *p* = 0.054, partial η^2^ = 0.11].

**Table 2:** Descriptive statistics for self-report psychological measures as a function of learning groups derived from hierarchical cluster analyses.

	**Cluster analysis 1 (all training data)**			**Cluster analysis 2 (Week 2 training data only)**		
	**Better learners (*n* = 14)**	**Intermediate learners (*n* = 15)**	**Poorer learners (*n* = 25)**	**Better learners (*n* = 14)**	**Intermediate learners (*n* = 13)**	**Poorer learners (*n* = 27)**
**PCL**						
Total score	38.86 (18.00)	41.67 (19.38)	22.20 (15.19)	43.64 (15.48)	35.39 (21.24)	24.19 (16.69)
Cluster B	2.00 (1.96)	2.13 (1.51)	1.00 (1.38)	2.50 (1.91)	1.62 (1.50)	1.07 (1.39)
Cluster C	2.64 (2.13)	3.00 (2.20)	0.88 (1.17)	3.21 (1.89)	2.31 (2.53)	1.07 (1.30)
Cluster D	2.29 (2.37)	2.07 (1.49)	0.92 (1.50)	2.57 (2.31)	1.39 (1.45)	1.19 (1.59)
Cluster E	2.64 (2.34)	2.60 (1.84)	1.32 (1.44)	2.93 (2.30)	2.15 (1.82)	1.48 (1.55)
PTSD+	9/14 (64%)	10/15 (67%)	6/25 (24%)	10/14 (71%)	7/13 (54%)	8/27 (30%)
**PHQ-8**						
Total score	12.64 (5.98)	11.73 (6.81)	6.60 (4.54)	13.79 (6.53)	10.62 (6.38)	6.93 (4.54)
MDD+	9/14 (64%)	9/15 (60%)	8/25 (32%)	10/14 (71%)	6/13 (46%)	10/27 (37%)
**AMBI**						
Total score	21.64 (5.18)	20.53 (7.33)	16.28 (5.89)	22.07 (5.71)	19.31 (6.95)	16.96 (6.21)
BI	13/14 (93%)	11/15 (73%)	13/25 (52%)	13/14 (93%)	9/13 (69%)	15/27 (56%)

*Learning groups include those generated from hierarchical cluster analyses using all training data (cluster analysis 1) and Week 2 training data only (cluster analysis 2)*.

It was notable that group stratification in CR expression was most pronounced during Week 2 testing, with minimal variability apparent between training blocks during this training period. Indeed, this corresponded to patterns observed for CR acquisition reported in our previous analysis for PTSD and mTBI veterans, where a greater degree of asymptotic performance was apparent across groups during Week 2. One question to emerge from these observations was whether greater discriminability of learning profiles (and thus greater discriminability in psychopathological symptoms) could be achieved using Week 2 performance as the only input in cluster analysis, as these data provided a higher degree of stability relative to Week 1 performance. Utilizing all nine training blocks as inputs in the previous cluster analysis effectively distinguished veterans on the basis of learning while also distinguishing Low Learners from Mid and High Learners on psychological measures. However, differences in psychological measures between the Mid and High Learners were not as apparent. To determine whether reducing inputs to Week 2 performance would better distinguish the psychological features of the more proficient learners, a second hierarchical cluster analysis was conducted. For comparison purposes with the previous analysis, a three-group solution was specified.

Although acquisition curves were similar to those of prior learning profiles produced using all training data from Week 1 and Week 2 (see Figure 2, right panel), tuning the clustering algorithm did appear to increase associations between the three learning profiles and PTSD, specifically. Among the High Learners, 10/14 (71%) were classified as PTSD^+^, whereas rates were lower among Mid (7/13; 54%) and Low Learners (8/27; 30%). By comparison, using the previous solution, 66% (10/15) of High Learners, 64% (9/14) of Mid Learners, and 24% (6/25) of Low Learners were considered PTSD^+^. Not surprisingly, this was also reflected in group differences in PCL total scores, with the highest scores among the High Learners (*M* = 43.64, *SD* = 15.48), followed by Mid (*M* = 35.39, *SD* = 21.24) and Low Learners (*M* = 24.19, *SD* = 16.69). Similarly, High Learners also produced higher PHQ-8 (*M* = 13.79, *SD* = 6.53) and AMBI (*M* = 22.07, *SD* = 5.71) total scores than Mid (PHQ-8: *M* = 10.61, *SD* = 6.38; AMBI: *M* = 19.31, *SD* = 6.95) and Low Learners (PHQ-8: *M* = 6.93, *SD* = 4.54; AMBI: *M* = 16.96, *SD* = 6.21). It is notable that 13/14 (93%) of the High Learners were classified as BI, compared to 9/13 (69%) of the Mid Learners, and 15/27 (56%) of the Low Learners.

When assessing PTSD symptomology specifically, significant between-groups effects were observed for Cluster B scores [*F*_(2,51)_ = 3.85, *p* = 0.028, partial η^2^ = 0.13] and Cluster C scores [*F*_(2,51)_ = 6.83, *p* = 0.002, partial η^2^ = 0.21]. There were not statistically significant differences for Cluster D [*F*_(2,51)_ = 2.93, *p* = 0.062, partial η^2^ = 0.10] nor Cluster E [*F*_(2,51)_ = 2.92, *p* = 0.063, partial η^2^ = 0.10] scores. Helmert contrasts revealed a significant differences between Poorer Learners and the higher proficiency learning groups for Clusters B (*p* = 0.025) and Cluster C (*p* = 0.001) scores; despite numerical differences in symptom severity, there were no significant differences between the Mid and High Learners in Cluster B (*p* = 0.148) nor Cluster C (*p* = 0.199) symptoms.

## Discussion

Consistent with prior research, acquisition of the classically conditioned eyeblink response was enhanced in veterans expressing current PTSD symptoms. A higher frequency of eyeblink CRs was recorded for PTSD+ veterans during both weekly training sessions, with the most dramatic between-groups difference in CR expression occurring during the Week 2 acquisition period. These results align with recent eyeblink conditioning work utilizing partial reinforcement training schedules in non-clinical civilian samples vulnerable to anxiety disorders (Allen et al., 2014) and PTSD+ active duty military (Handy et al., 2018), while also elaborating on the stability of acquired responding under these conditions over time. Specifically, facilitative effects in PTSD were apparent 7 days following the initial training period. Although there was some indication that history of mTBI may modulate CR expression in PTSD, these effects were limited to Week 2 observations. Finally, in an exploratory aim, an unsupervised machine learning methodology was used to subtype participants on the basis of eyeblink conditioning performance and found that the resultant learning groups significantly differed on self-reported psychiatric symptoms and anxiety-vulnerable personality characteristics. Among the most proficient learners, there was greater incidence of clinically significant PTSD and MDD symptomology as well as higher scores on a measure of BI. These findings are discussed further below.

### Learning Biases in Eyeblink Conditioning

Positively biased associative learning in veteran PTSD+ participants supports the hypothesized learning diathesis model of anxiety and stress disorders (Caulfield et al., 2013; Handy et al., 2018; Allen et al., 2019). To review, this theoretical model posits inherent differences in associative learning underlie the development and maintenance of anxiety-related symptomology—particularly the over-expression of avoidance behaviors common to PTSD (Allen et al., 2019). Central to this learning diathesis is abnormal functioning of the cerebellum, which has garnered increased appreciation in recent years for its contributions to higher order cognitive processes related to emotion and anxiety. Previous studies have reported augmented cerebellar reactivity following exposure to personalized, script-driven imagery designed to be stress-inducing (Jastreboff et al., 2011; Seo et al., 2011). Similarly, increases in cerebellar reactivity to trauma-related cues has been reported in earthquake survivors expressing symptoms of PTSD (Yang et al., 2004), survivors of workplace accidents (Ke et al., 2016), combat-exposed veterans with PTSD (Bremner et al., 2017), in addition to reports of significant activation in response to subliminal processing of trauma-related words in PTSD patients (Rabellino et al., 2016). Cerebellar hyper-reactivity to stress-related cues may reflect, in part, greater sensitivity to uncertainty in the environment, particularly for cues and contingencies that may be perceived to signal danger. A causal link between the cerebellum and aberrant probabilistic risk assessments has been argued in relation to the over-expression of defensive stress reactions (e.g., avoidance, withdrawal) characteristic of PTSD and other anxiety disorders (Rabellino et al., 2016; Lanius et al., 2017).

As discussed previously, eyeblink classical conditioning serves as a useful model paradigm for examining deviations in cerebellar processing in PTSD, and has frequently been employed to study the behavioral and neurobiological features of other neurological and psychiatric conditions. Although evidence of enhanced learning in PTSD+ veterans was apparent in a prior study (Myers et al., 2012a), the degree of distinctiveness was relatively small and required large numbers of veterans to be sensitive to group differences. Partial reinforcement training schedules were tested in subsequent studies in an attempt to accentuate group differences in learning, in part by exploiting inherent differences in sensitivity to uncertainty theorized to exist in anxiety-prone and clinical populations (Allen et al., 2014; Handy et al., 2018). In line with these previous investigations, in the current study suboptimal learning conditions imposed by the 50% CR partial reinforcement schedule facilitated acquisition in PTSD+ veterans, which was quite pronounced and provided exceptional separation between symptom groups. Given that a stereotypic learning effect has been shown to generalize across several unique cohorts of participants, ranging from undergraduate students to active duty military and veteran populations, these results strongly suggest future studies impose similar learning conditions to maximize the utility of the eyeblink conditioning paradigm in the study of stress and anxiety.

An examination of Week 2 training data revealed conditioned performance was largely conserved across groups after 7 days (see Figure 1). There is a dearth of empirical studies on retention of conditioned responding in delay eyeblink conditioning in humans, although a small literature exists detailing development of associative learning processes in infants (Ivkovich et al., 1999; Klaflin et al., 2002). To our knowledge, no study has examined the stability of conditioned responding over multiple weekly training sessions within psychiatric groups. The fact that participants across symptom groups maintained asymptotic performance between sessions suggests that delay conditioning protocols could serve as useful vehicles for monitoring functional changes associated with treatment over time. For example, while a great many benefit from current practices a significant number of those expressing PTSD symptoms are treatment resistant (Steenkamp et al., 2015). Eyeblink conditioning may serve as a biomarker of treatment resistance and a target of pharmacological and psychobehavioral interventions.

### Modulatory Influences of Lifetime History of mTBI on Learning

With regard to moderation of eyeblink conditioning performance based on lifetime history of mTBI, results were mixed. Although there was no indication acquisition was influenced by mTBI history in Week 1 performance, a significant interaction between PTSD and mTBI during Week 2 did suggest differences in CR expression among PTSD+ veterans with prior head injuries. As illustrated in Figure 1, PTSD+ veterans without a history of mTBI expressed the eyeblink CR during Week 2 at a much higher frequency than PTSD+ veterans with a previous head injury. No statistically significant differences were apparent when comparing PTSD– veterans with and without a history of mTBI, although those with a prior mTBI incident did produce a numerically higher number of CRs during Week 2 training. This observation runs counter to the hypothesized direction of the mTBI effect in eyeblink conditioning. The sample size of veterans classified as PTSD–/mTBI– was relatively small, however (*n* = 8). It is also worth noting that, although there is mounting evidence to suggest deleterious effects of prior head injury on cerebellar processes, it is unlikely that all mTBI incidents result in cerebellar injury or dysfunction. To the degree historical mTBI incidents resulted in cerebellar damage or associated dysfunction is not clear in the current sample. Reported cause of injury in those with lifetime history of mTBI was quite diverse as well and included experience with mechanical injury and service-related blast exposure. As reviewed previously, the cerebellum may be particularly vulnerable to blast injury and exposures (Mac Donald et al., 2013; Meabon et al., 2016) making this injury group more likely to demonstrate impairments in cerebellar tasks, such as eyeblink classical conditioning. Unfortunately, sample size considerations limited a deeper exploration of injury mechanism in relation to eyeblink conditioning performance.

### Predicting Symptom Groups Using Conditioned Eyeblink Responses

A final contribution of the current study was the novel use of cluster analysis to construct distinctive learning profiles based on the rate and frequency of CR expression. Although data clustering methods have been applied more frequently in recent years to subtype and stratify psychiatric conditions, applications of these approaches within the PTSD literature are limited (for a review see Dollfus et al., 1996; Marquand et al., 2016). To our knowledge this study is the first to explore clustering using eyeblink conditioning data as inputs for group stratification. That the resultant learning profiles effectively stratified veterans into clinically meaningful subgroups is notable, given that the algorithm relied solely on individual training performance for data partitioning and was naïve to the results of self-report measures of personality and symptomology. By limiting inputs for cluster analysis to Week 2 conditioning data, in which a greater degree of asymptotic performance was apparent across participants, greater sensitivity to PTSD-related symptomology within learning profiles was achieved. The dramatic disparity in CR expression was particularly evident between High and Low Learners. Incidentally, a significantly greater number of PTSD+ veterans were identified within the High Learners. Further, consistent with expectations from the learning diathesis model of PTSD, Mid and High Learners also expressed a greater degree of Cluster C symptomology, reflecting avoidance-related behaviors on the PCL-5. Inherent associative learning biases are argued to contribute to the development of pathological avoidance behaviors in anxiety and stress disorders.

These results also suggest eyeblink conditioning data to be more informative for identifying stress-related vulnerability or current symptomology following multiple training sessions wherein asymptotic performance is more likely. A week was used in the current study, although it is certainly possible shorter inter-session intervals could be sufficient. This data-driven approach to examining psychopathology aligns with the National Institute of Mental Health Research Domain Criteria (RDoC) initiative to utilize behavioral and physiological indicators in the characterization of psychiatric disorders and further supports the potential use of eyeblink classical conditioning as a clinical identification tool sensitive to anxiety-related symptomology. Eyeblink conditioning may find utility as a component of a screening process for at-risk populations. While the clustering algorithm of eyeblink conditioning identified those expressing PTSD symptoms at the group level, further refinements are necessary to have utility at the individual level.

### Study Limitations and Conclusions

There were a few noteworthy limitations of the current investigation. First, sample sizes for subgroup analyses were modest and often unbalanced. Participants were not pre-screened prior to completing study procedures and were grouped later based on self-report measures completed as part of the study. To the degree that responses indexed by self-report measures reflected clinically meaningful symptoms is not clear, although several participants mentioned that they were currently seeking treatment or had previously sought treatment for stress-related disorders. Related to this, and as mentioned previously, sample size considerations limited our ability to be more granular in our treatment of mTBI, particularly with regard to mechanism of injury, number of incidents, etc. Finally, the cross-sectional nature of the study limited our ability to causally tie associative learning to anxiety and PTSD, despite the strong supporting evidence these results provide for learning as a diathesis. Future work should assess to the degree associative learning biases are predictive of later psychopathology, emphasizing prospective assessments of learning and longitudinal observation to better define causal relationships.

In conclusion, the current study advances a nascent learning diathesis model of stress and anxiety by demonstrating abnormal associative learning behavior in veterans self-reporting symptoms of PTSD. Delay eyeblink classical conditioning has served as a powerful model paradigm for examining inherent learning biases in clinical and non-clinical populations, highlighting deviations in cerebellar-based learning that may contribute to development and maintenance of defensive behaviors in anxiety, such as avoidance. These results support the continued use of sub-optimal learning conditions, such as partial reinforcement training schedules, to achieve heightened sensitivity to group differences in acquisition. The 50% CS partial reinforcement schedule used in the current study has proven particularly useful for identifying facilitated acquisition effects in PTSD and anxiety-prone personality types. Moreover, the stability of conditioned responding over weekly training sessions strongly supports the future use of this associative learning paradigm in longitudinal work utilizing repeated measures, e.g., surveying learning in parallel with symptom expression over the course of treatment for anxiety and stress disorders. The current study also provided a cursory examination of the modulatory influences of a lifetime history of mTBI on cerebellar-based learning in PTSD. Less frequent CR expression in PTSD+ veterans with a history of mTBI during Week 2 was suggestive, and clearly warrants further examination. In sum, these results support the continued investigation of the conditioned eyeblink response as a behavioral indicator of stress-related psychopathology.

## Data Availability Statement

The raw data supporting the conclusions of this article will be made available by the authors, without undue reservation.

## Ethics Statement

The studies involving human participants were reviewed and approved by Syracuse/Bath/Canandaigua VA Medical Centers Institutional Review Board. The patients/participants provided their written informed consent to participate in this study.

## Author Contributions

JH, WW, and RS designed the study, wrote the protocol, and wrote the first draft of the manuscript. AH, LS, and JH collected, processed, and prepared the data for analysis. JH undertook the statistical analysis. All the authors commented on the manuscript, contributed to, and have approved the final manuscript.

## Conflict of Interest

The authors declare that the research was conducted in the absence of any commercial or financial relationships that could be construed as a potential conflict of interest.

## References

[B1] Allen M. T. Handy J. D. Miller D. P. Servatius R. J. (2019). Avoidance learning and classical eyeblink conditioning as model systems to explore a learning diathesis model of PTSD. Neurosci. Biobehav. Rev. 100, 370–386. 10.1016/j.neubiorev.2019.03.003 30952323

[B2] Allen M. T. Myers C. E. Servatius R. J. (2014). Avoidance prone individuals self reporting behavioral inhibition exhibit facilitated acquisition and altered extinction of conditioned eyeblinks with partial reinforcement schedules. Front. Behav. Neurosci. 8:347. 10.3389/fnbeh.2014.00347 25339877 PMC4186341

[B3] Allen M. T. Myers C. E. Servatius R. J. (2016). Uncertainty of trial timing enhances acquisition of conditioned eyeblinks in anxiety vulnerable individuals. Behav. Brain Res. 304, 86–91. 10.1016/j.bbr.2016.02.007 26873040

[B4] Bazarian J. J. Mcclung J. Shah M. N. Ting Cheng Y. Flesher W. Kraus J. (2005). Mild traumatic brain injury in the United States, 1998–2000. Brain Injury 19, 85–91. 10.1080/02699050410001720158 15841752

[B5] Beck K. D. Brennan F. X. Servatius R. J. (2002). Effects of stress on nonassociative learning processes in male and female rats. Integr. Physiol. Behav. Sci. 37, 128–139. 10.1007/BF02688825 12186307

[B6] Biederman J. Rosenbaum J. F. Hirshfeld D. R. Faraone S. V. Bolduc E. A. Gersten M.. (1990). Psychiatric correlates of behavioral inhibition in young children of parents with and without psychiatric disorders. Arch. Gen. Psychiatry 47, 21–26. 10.1001/archpsyc.1990.01810130023004 2294852

[B7] Bremner J. D. Mishra S. Campanella C. Shah M. Kasher N. Evans S.. (2017). A pilot study of the effects of mindfulness-based stress reduction on post-traumatic stress disorder symptoms and brain response to traumatic reminders of combat in Operation Enduring Freedom/Operation Iraqi Freedom combat veterans with post traumatic stress disorder. Front. Psychiatry. 8:157. 10.3389/fpsyt.2017.00157 28890702 PMC5574875

[B8] Carlson K. F. Meis L. A. Jensen A. C. Simon A. B. Gravely A. A. Taylor B. C.. (2012). Caregiver reports of subsequent injuries among veterans with traumatic brain injury after discharge from inpatient polytrauma rehabilitation programs. J. Head Trauma Rehabil. 27, 14–25. 10.1097/HTR.0b013e318236bd86 22218200

[B9] Caulfield M. D. McAuley J. D. Servatius R. J. (2013). Facilitated acquisition of eyeblink conditioning in those vulnerable to anxiety disorders. Front. Hum. Neurosci. 7:348. 10.3389/fnhum.2013.00348 23847516 PMC3701872

[B10] Caulfield M. D. VanMeenen K. M. Servatius R. J. (2015). Facilitated acquisition of standard but not long delay classical eyeblink conditioning in behaviorally inhibited adolescents. Behav. Brain Res. 278, 476–481. 10.1016/j.bbr.2014.10.027 25447303

[B11] Charrad M. Ghazzali N. Boiteau V. Niknafs A. (2014). NbClust: An R package for determining the relevant number of clusters in a data set. J. Stat. Softw. 61, 1–36. 10.18637/jss.v061.i06

[B12] Cheng D. T. Disterhoft J. F. Power J. M. Ellis D. A. Desmond J. E. (2008). Neural substrates underlying human delay and trace eyeblink conditioning. Proc. Natl. Acad. Sci. U.S.A. 105, 8108–8113. 10.1073/pnas.0800374105 18523017 PMC2430367

[B13] Degnan K. A. Fox N. A. (2007). Behavioral inhibition and anxiety disorders: multiple levels of a resilience process. Dev. Psychopathol. 19, 729–746. 10.1017/S0954579407000363 17705900

[B14] Dollfus S. Everitt B. Ribeyre J. M. Assouly-Besse F. Sharp C. Petit M. (1996). Identifying subtypes of schizophrenia by cluster analyses. Schizophr. Bull. 22, 545–555. 10.1093/schbul/22.3.545 8873304

[B15] Edwards C. R. Newman S. Bismark A. Skosnik P. D. O'Donnell B. F. Shekhar A.. (2008). Cerebellum volume and eyeblink conditioning in schizophrenia. Psychiatry Res. 162, 185–194. 10.1016/j.pscychresns.2007.06.001 18222655 PMC2366060

[B16] Freeman J. H. Steinmetz A. B. (2011). Neural circuitry and plasticity mechanisms underlying delay eyeblink conditioning. Learn. Mem. 18, 666–677. 10.1101/lm.2023011 21969489 PMC3861981

[B17] Gale S. D. Baxter L. Roundy N. Johnson S. C. (2005). Traumatic brain injury and grey matter concentration: a preliminary voxel based morphometry study. J. Neurol. Neurosurg. Psychiatry 76, 984–988. 10.1136/jnnp.2004.036210 15965207 PMC1739692

[B18] Gladstone G. Parker G. (2005). Measuring a behaviorally inhibited temperament style: development and initial validation of new self-report measures. Psychiatry Res. 135, 133–143. 10.1016/j.psychres.2005.03.005 15922458

[B19] Greer T. L. Thompson L. T. (2017). Editorial: eyeblink classical conditioning in psychiatric conditions: novel uses for a classic paradigm. Front. Psychiatry 8:48. 10.3389/fpsyt.2017.00048 28396641 PMC5367055

[B20] Greer T. L. Trivedi M. H. Thompson L. T. (2005). Impaired delay and trace eyeblink conditioning performance in major depressive disorder. J. Affect. Disord. 86, 235–245. 10.1016/j.jad.2005.02.006 15935243

[B21] Gudino O. G. (2013). Behavioral inhibition and risk for posttraumatic stress symptoms in Latino children exposed to violence. J. Abnorm. Child Psychol. 41, 983–992. 10.1007/s10802-013-9731-2 23494527 PMC3709003

[B22] Handy J. D. Avcu P. Ko N. Ortiz A. Doria M. J. Servatius R. J. (2018). Facilitated acquisition of the classically conditioned eyeblink response in active duty military expressing posttraumatic stress disorder symptoms. Behav. Brain Res. 339, 106–113. 10.1016/j.bbr.2017.11.014 29154809

[B23] Hirshfeld D. R. Rosenbaum J. F. Biederman J. Bolduc E. A. Faraone S. V. Snidman N.. (1992). Stable behavioral inhibition and its association with anxiety disorder. J. Am. Acad. Child Adolesc. Psychiatry 31, 103–111. 10.1097/00004583-199201000-00016 1537760

[B24] Hoge C. W. McGurk D. Thomas J. L. Cox A. L. Engel C. C. Castro C. A. (2008). Mild traumatic brain injury in US soldiers returning from Iraq. N. Engl. J. Med. 358, 453–463. 10.1056/NEJMoa072972 18234750

[B25] Holloway J. L. Allen M. T. Myers C. E. Servatius R. J. (2014). Behaviorally inhibited individuals demonstrate significantly enhanced conditioned response acquisition under non-optimal learning conditions. Behav. Brain Res. 261, 49–55. 10.1016/j.bbr.2013.10.041 24275381

[B26] Holloway J. L. Trivedi P. Myers C. E. Servatius R. J. (2012). Enhanced conditioned eyeblink response acquisition and proactive interference in anxiety vulnerable individuals. Front. Behav. Neurosci. 6:76. 10.3389/fnbeh.2012.00076 23162449 PMC3499707

[B27] Ivkovich D. Collins K. L. Eckerman C. O. Krasnegor N. A. Stanton M. E. (1999). Classical delay eyeblink conditioning in 4-and 5-month-old human infants. Psychol. Sci. 10, 4–8. 10.1111/1467-9280.00097

[B28] Jastreboff A. M. Potenza M. N. Lacadie C. Hong K. A. Sherwin R. S. Sinha R. (2011). Body mass index, metabolic factors, and striatal activation during stressful and neutral-relaxing states: an FMRI study. Neuropsychopharmacology 36, 627–637. 10.1038/npp.2010.194 21048702 PMC3055687

[B29] Kagan J. Reznick J. S. Snidman N. (1987). The physiology and psychology of behavioral inhibition in children. Child Dev. 58, 1459–1473. 10.2307/1130685 3691195

[B30] Ke J. Zhang L. Qi R. Li W. Hou C. Zhong Y.. (2016). A longitudinal fMRI investigation in acute post-traumatic stress disorder (PTSD). Acta Radiol. 57, 1387–1395. 10.1177/0284185115585848 25995310

[B31] Klaflin D. I. Stanton M. E. Herbert J. Greer J. Eckerman C. O. (2002). Effect of delay interval on classical eyeblink conditioning in 5-month-old human infants. Dev. Psychobiol. 41, 329–340. 10.1002/dev.10050 12430157

[B32] Kroenke K. Strine T. W. Spitzer R. L. Williams J. B. Berry J. T. Mokdad A. H. (2009). The PHQ-8 as a measure of current depression in the general population. J. Affect. Disord. 114, 163–173. 10.1016/j.jad.2008.06.026 18752852

[B33] Lanius R. A. Rabellino D. Boyd J. E. Harricharan S. Frewen P. A. McKinnon M. C. (2017). The innate alarm system in PTSD: conscious and subconscious processing of threat. Curr. Opin. Psychol. 14, 109–115. 10.1016/j.copsyc.2016.11.006 28813307

[B34] Lee D. J. Bovin M. J. Weathers F. W. Palmieri P. A. Schnurr P. P. Sloan D. M.. (2019). Latent factor structure of DSM-5 posttraumatic stress disorder: evaluation of method variance and construct validity of novel symptom clusters. Psychol. Assess. 31, 46–58. 10.1037/pas0000642 30113182 PMC6312504

[B35] Mac Donald C. Johnson A. Cooper D. Malone T. Sorrell J. Shimony J.. (2013). Cerebellar white matter abnormalities following primary blast injury in US military personnel. PLoS ONE 8:e55823. 10.1371/journal.pone.0055823 23409052 PMC3567000

[B36] Mac Donald C. L. Johnson A. M. Cooper D. Nelson E. C. Werner N. J. Shimony J. S.. (2011). Detection of blast-related traumatic brain injury in US military personnel. N. Engl. J. Med. 364, 2091–2100. 10.1056/NEJMoa1008069 21631321 PMC3146351

[B37] Marquand A. F. Wolfers T. Mennes M. Buitelaar J. Beckmann C. F. (2016). Beyond lumping and splitting: a review of computational approaches for stratifying psychiatric disorders. Biol. Psychiatry. Cogn. Neurosci. Neuroimaging. 1, 433–447. 10.1016/j.bpsc.2016.04.002 27642641 PMC5013873

[B38] Meabon J. S. Huber B. R. Cross D. J. Richards T. L. Minoshima S. Pagulayan K. F.. (2016). Repetitive blast exposure in mice and combat veterans causes persistent cerebellar dysfunction. Sci. Transl. Med. 8:321ra6. 10.1126/scitranslmed.aaa9585 26764157

[B39] Miller M. J. Chen N. Li L. Tom B. Weiss C. Disterhoft J. F.. (2003). fMRI of the conscious rabbit during unilateral classical eyeblink conditioning reveals bilateral cerebellar activation. J. Neurosci. 23, 11753–11758. 10.1523/JNEUROSCI.23-37-11753.2003 14684877 PMC6740957

[B40] Morgan B. E. (2006). Behavioral inhibition: a neurobiological perspective. Curr. Psychiatry Rep. 8, 270–278. 10.1007/s11920-006-0062-7 16879790

[B41] Myers C. E. VanMeenen K. M. McAuley J. D. Beck K. D. Pang K. C. Servatius R. J. (2012b). Behaviorally-inhibited temperament is associated with severity of PTSD symptoms and faster eyeblink conditioning in veterans. Stress 15:31. 10.3109/10253890.2011.578184 21790343 PMC3364604

[B42] Myers C. E. VanMeenen K. M. Servatius R. J. (2012a). Behavioral inhibition and PTSD symptoms in veterans. Psychiatry Res. 196, 271–276. 10.1016/j.psychres.2011.11.015 22397911 PMC3361537

[B43] Peskind E. R. Petrie E. C. Cross D. J. Pagulayan K. McCraw K. Hoff D.. (2011). Cerebrocerebellar hypometabolism associated with repetitive blast exposure mild traumatic brain injury in 12 Iraq war Veterans with persistent post-concussive symptoms. Neuroimage 54, S76–S82. 10.1016/j.neuroimage.2010.04.008 20385245 PMC3264671

[B44] Petrie E. C. Cross D. J. Yarnykh V. L. Richards T. Martin N. M. Pagulayan K.. (2014). Neuroimaging, behavioral, and psychological sequelae of repetitive combined blast/impact mild traumatic brain injury in Iraq and Afghanistan war veterans. J. Neurotrauma 31, 425–436. 10.1089/neu.2013.2952 24102309 PMC3934596

[B45] R Core Team (2020). R: A Language and Environment for Statistical Computing. Vienna: R Foundation for Statistical Computing. Available online at: https://www.R-project.org/

[B46] Rabellino D. Densmore M. Frewen P. A. Théberge J. McKinnon M. C. Lanius R. A. (2016). Aberrant functional connectivity of the amygdala complexes in PTSD during conscious and subconscious processing of trauma-related stimuli. PloS one 11:e0163097. 10.1371/journal.pone.0163097 27631496 PMC5025207

[B47] Rampello L. Casolla B. Rampello L. Pignatelli M. Battaglia G. Gradini R.. (2011). The conditioned eyeblink reflex: a potential tool for the detection of cerebellar dysfunction in multiple sclerosis. Mult. Scler. J. 17, 1155–1161. 10.1177/1352458511406311 21613334

[B48] Sato M. Chang E. Igarashi T. Noble L. J. (2001). Neuronal injury and loss after traumatic brain injury: time course and regional variability. Brain Res. 917, 45–54. 10.1016/S0006-8993(01)02905-5 11602228

[B49] Schwab K. A. Gudmudsson L. S. Lew H. L. (2015). Long-term functional outcomes of traumatic brain injury. Handb. Clin. Neurol. 128, 649–659. 10.1016/B978-0-444-63521-1.00040-6 25701912

[B50] Seo D. Jia Z. Lacadie C. M. Tsou K. A. Bergquist K. Sinha R. (2011). Sex differences in neural responses to stress and alcohol context cues. Hum. Brain Mapp. 32, 1998–2013. 10.1002/hbm.21165 21162046 PMC3236497

[B51] Servatius R. J. (2000). Eyeblink conditioning in the freely moving rat: square-wave stimulation as the unconditioned stimulus. J. Neurosci. Methods 102, 35–42. 10.1016/S0165-0270(00)00276-4 11000409

[B52] Servatius R. J. (2016). Editorial: avoidance: from basic science to psychopathology. Front. Behav. Neurosci. 10:15. 10.3389/fnbeh.2016.00015 26903831 PMC4751251

[B53] Servatius R. J. Beck K. D. (2003). Facilitated acquisition of the classically conditioned eyeblink response in male rats after systemic IL-1beta. Integr. Physiol. Behav. Sci. 38, 169–178. 10.1007/BF02688851 15070080

[B54] Servatius R. J. Beck K. D. Moldow R. L. Salameh G. Tumminello T. P. Short K. R. (2005). A stress-induced anxious state in male rats: corticotropin-releasing hormone induces persistent changes in associative learning and startle reactivity. Biol. Psychiatry 57, 865–872. 10.1016/j.biopsych.2005.01.015 15820707

[B55] Servatius R. J. Handy J. D. Doria M. J. Myers C. E. Marx C. E. Lipsky R.. (2017). Stress-related mental health symptoms in Coast Guard: incidence, vulnerability, and neurocognitive performance. Front. Psychol. 8:1513. 10.3389/fpsyg.2017.01513 28959220 PMC5603677

[B56] Servatius R. J. Shors T. J. (1994). Exposure to inescapable stress persistently facilitates associative and nonassociative learning in rats. Behav. Neurosci. 108, 1101–1106. 10.1037/0735-7044.108.6.1101 7893402

[B57] Shors T. J. Servatius R. J. (1997). The contribution of stressor intensity, duration, and context to the stress-induced facilitation of associative learning. Neurobiol. Learn. Mem. 68, 92–96. 10.1006/nlme.1997.3763 9195594

[B58] Solomon P. R. Beal M. F. Pendlebury W. W. (1988). Age-related disruption of classical conditioning: A model systems approach to memory disorders. Neurobiol. Aging 9, 535–546. 10.1016/S0197-4580(88)80110-6 3062464

[B59] Steenkamp M. M. Litz B. T. Hogue C. W. Marmar C. R. (2015). Psychotherapy for military-related PTSD: a review of randomized clinical trials. JAMA 314, 489–500. 10.1001/jama.2015.8370 26241600

[B60] Steinmetz J. E. Tracy J. A. Green J. T. (2001). Classical eyeblink conditioning: clinical models and applications. Integr. Physiol. Behav. Sci. 36, 220–238. 10.1007/BF02734095 11777017

[B61] Terrio H. P. Nelson L. A. Betthauser L. M. Harwood J. E. Brenner L. A. (2011). Postdeployment traumatic brain injury screening questions: sensitivity, specificity, and predictive values in returning soldiers. Rehabil. Psychol. 56:26 10.1037/a0022685 21401283

[B62] Weathers F. W. Blake D. D. Schnurr P. P. Kaloupek D. G. Marx B. P. Keane T. M. (2013). The Life Events Checklist for DSM-5 (LEC-5). National Center for PTSD. Available online at: www.ptsd.va.gov

[B63] Woodruff-Pak D. S. Thompson R. F. (1988). Classical conditioning of the eyeblink response in the delay paradigm in adults aged 18–83 years. Psychol. Aging 3, 219–229. 10.1037//0882-7974.3.3.219 3268262

[B64] Wright W. G. Handy J. D. Avcu P. Ortiz A. Haran F. J. Doria M. (2018). Healthy active duty military with lifetime experience of mild traumatic brain injury exhibits subtle deficits in sensory reactivity and sensory integration during static balance. Milit. Med. 183, 313–320. 10.1093/milmed/usx182 29635588

[B65] Yang P. Wu M. T. Hsu C. C. Ker J. H. (2004). Evidence of early neurobiological alternations in adolescents with posttraumatic stress disorder: a functional MRI study. Neurosci. Lett. 370, 13–18. 10.1016/j.neulet.2004.07.033 15489009

